# Recurrent IVF failure and hereditary thrombophilia 

**Published:** 2014-07

**Authors:** Leila Safdarian, Zahra Najmi, Ashraf Aleyasin, Marzieh Aghahosseini, Mandana Rashidi, Sara Asadollah

**Affiliations:** 1 *Department of Infertility, Shariati Hospital, Tehran University of Medical Sciences, Tehran, Iran.*; 2 *Department of Infertility, Shahid Akbar-abadi Hospital, Iran University of Medical Sciences, Tehran, Iran.*

**Keywords:** *Recurrent implantation failure*, *In vitrofertilization*, *Hereditary thrombophilia*

## Abstract

**Background:** The largest percentage of failed invitro fertilization (IVF (cycles, are due to lack of implantation. As hereditary thrombophilia can cause in placentation failure, it may have a role in recurrent IVF failure.

**Objective:** Aim of this case-control study was to determine whether hereditary thrombophilia is more prevalent in women with recurrent IVF failures.

**Materials and Methods: **Case group comprised 96 infertile women, with a history of recurrent IVF failure. Control group was comprised of 95 healthy women with proven fertility who had conceived spontaneously. All participants were assessed for the presence of inherited thrombophilias including: factor V Leiden, methilen tetrahydrofolate reductase (MTHFR) mutation, prothrombin mutation, homocystein level, protein S and C deficiency, antithrombin III (AT-III) deficiency and plasminogen activator inhibitor-1 (PAI-1) mutation. Presence of thrombophilia was compared between groups.

**Results: ** Having at least one thrombophilia known as a risk factor for recurrent IVF failure (95% CI=1.74-5.70, OR=3.15, p=0.00). Mutation of factor V Leiden (95% CI=1.26-10.27, OR=3.06, P=0.01) and homozygote form of MTHFR mutation (95% CI=1.55-97.86, OR=12.33, p=0.05) were also risk factors for recurrent IVF failure. However, we could not find significant difference in other inherited thrombophilia’s.

**Conclusion:** Inherited thrombophilia is more prevalent in women with recurrent IVF failure compared with healthy women. Having at least one thrombophilia, mutation of factor V Leiden and homozygote form of MTHFR mutation were risk factors for recurrent IVF failure.

## Introduction

Despite the multiple embryo transfer in the majority of infertility centers, only one third of all in vitro fertilization (IVF) cycles reach clinically achieved pregnancy and the majority of them still fail (1). The largest percentage of failed IVF cycles, are due to lack of implantation. In some patients, implantation failure occurs repeatedly (2). Recurrent IVF failure commonly defines as Failure to achieve a pregnancy following 2-6 IVF cycles, in which more than 10 high-grade embryos were transferred into the uterus (3).

Embryo quality and endometrial receptivity are two significant factors that believed to be the key points in implantation. The possible causes of repeated embryo implantation failure have been widely investigated. The most identified causes are decreased endometrial receptivity, embryonic defects and factors with combined effects (4, 5). Suggested methods for investigation and treatment of recurrent IVF failure is 1) improving endometrial receptivity, 2) treatment of the embryos, and 3) multifactorial treatment options (4).

Recently, the role of hereditary thrombophilia in recurrent IVF failure is implicated. The association between thrombophilia and recurrent pregnancy loss or poor pregnancy outcome is well known. It may act by impairing the initial vascularization process occurring at implantation, which is necessary for a successful pregnancy (6-9). However, there are limited data on the association between thrombophilia, hereditary or acquired, with female infertility and IVF failure (7, 8, 10-12) especially in our population, Iran. Present study was conducted to determine the incidence of undiagnosed thrombophilia factors among the population of infertile women referring to Shariati Infertility Center; and to investigate whether hereditary thrombophilia is more prevalent in woman with recurrent IVF failures.

## Materials and methods

With approval of the ethics committee of Tehran University of Medical Sciences and after obtaining written consent from all participants, this prospective study was conducted between 2007 and 2011 in Shariati Hospital IVF Center, Tehran, Iran. Case group was comprised 96 infertile women, aged 20-40 years old, with a history of recurrent IVF failure. Recurrent IVF failure was defined as at least three consecutive failed IVF cycles. Failed IVF cycle was defined as failure to achieve clinical pregnancy in a cycle in which at least three good quality embryos (grade I or II) were transferred. Indications for IVF were male factor, ovulatory factor, tubal factor and unexplained infertility. In our center all fertilizations are performed by intra cytoplasmic sperm injection (ICSI).

Control group was comprised of 95 healthy women with proven fertility, matched for age, with no history of thrombo-embolic events, who had conceived spontaneously and had at least one uneventful pregnancy, without any complication (such as preeclampsia, intrauterine growth restriction and intrauterine fetal death). They were selected among women who were delivered in our tertiary hospital during the research period. Smokers, women of known anti phospholipid syndrome, women with history of thrombo-embolic events, diabetes, thyroid diseases, collagen-vascular diseases, uterine myoma, endometriosis, hydrosalpinx, and uterine cavity abnormalities were excluded from the study. To omit the probable effect of steroids on protein S, participants were chosen among patients who have not administered any hormonal agents (estrogen and combined contraceptives) in recent two months. As folic acid, vitamin B6 and B12 may alter the homocystein level, patients also have not administered folic acid or multivitamin supplements in recent two months. 

All participants were screened for antiphospholipid antibodies and they excluded from study if they had positive results. Others were assessed for the presence of inherited thrombophilia’s including: factor V Leiden, methylene tetrahydrofolate reductase (MTHFR) C677T mutation, prothrombin 20210A mutation, homocystein level, protein S and C deficiency, antithrombin III (AT-III) deficiency, and plasminogen activator inhibitor-1 (PAI-1) mutation. All lab tests were performed and repeated after 6 weeks in cases of abnormal findings, in same laboratory, using PCR method for factor V Leiden, chromogenic method for antithrombin 3, and ELISA method for prothrombin 20210G, protein S, protein c and PAI_1. 

**Statistical analysis**

Numerical variables were reported as mean±SD. We used independent sample t-test and Chi-square test to compare quantitative and qualitative variables, respectively. To evaluate whether inherited thrombophilia’s could predict the recurrent IVF failure, we used logistic regression analysis. Univariable analysis was performed, in which odds ratio (ORS) and 95% confidence intervals (95% CIs) were calculated. P-value ≤0.05 was considered to be statistically significant. All analyses were performed using SPSS software (Statistical Package for the Social Sciences, version 14.0, SPSS Inc, Chicago, Illinois, USA).

## Results

The analysis was performed on the complete data of 191 patients (96 in case group and 95 in control group). Mean age of the study population was 34.59±5.11, which was comparable between groups. Case group comprised primary and secondary infertile couples in 67.7% and 32.2%, respectively. Mean duration of infertility was 8.7±3.6 years. The most prevalent infertility cause was unexplained infertility in 39 (40.6%) patients. Other causes were male factor in 22 (22.9%), ovulatory factor in 21 (21.9%), and tubal factor in 14 (14.6%) patients (Table I). As shown in table II, the prevalence of thrombophilia was not significantly different in infertility groups with different etiologies. Fifty nine patients in case group (61.5%) had at least one inherited thrombophilia compared with 31 women (32.61%) in control group. 

When the impact of inherited thrombophilia on recurrent IVF failure, was evaluated by regression, a significant association was observed and having at least one thrombophilia known as a risk factor for recurrent IVF failure (95% CI=1.74-5.70, OR=3.15, p=0.000) (Table III). Factor V Leiden mutation (found in 11%) and MTHFRC677T mutation (homozygote form found in 6.3% and heterozygote form in 11.5%) were the most common inherited thrombophilia’s. The prevalence of inherited thrombophilia’s is displayed in Table I. 

Prevalence of factor V Leiden mutation and MTHFR C677T mutation in recurrent IVF failure group was significantly higher compared to controls (p<0.05). Mutation of factor V Leiden and homozygote form of MTHFR C677T mutation were risk factors for recurrent IVF failure. However, we could not find this difference in other inherited thrombophilia’s including prothrombin mutation, hyperhomocysteinemia, protein S and C deficiency, AT-III deficiency, and PAI-1 mutation. As shown in table III, we had no case of PAI-1 mutation in healthy group.

**Table I:** Characteristics of the infertile study group (N=96)

Characteristics		
Infertility duration (years, mean± SD)		8.71±3.65 (2-16)
Infertility type (n, %)		
	Primary	65 (67.7%)
	Secondary	31 (32.2%)
Infertility cause (n, %)		
	Ovarian factor	21 (21.9%)
	Tubal factor	14 (14.6%)
	Male factor	22 (22.9%)
	Unexplained	39 (40.6%)

**Table II:** Inherited thrombophilia and infertility cause

	**Thrombophilia positive [n (%)]**	**Thrombophilia negative [n (%)]**	**p-value**	**OR**
Ovarian factor	12 (57.1)	9 (42.9)	0.80	1.14 (0.53-2.45)
Tubal factor	8 (57.1)	6 (42.9)	0.77	1.14 (0.43-3.03)
Male factor	12 (54.5)	10 (45.5)	0.62	1.27 (0.61-2.64)
Unexplained	26 (66.7)	13 (33.3)	0.396	1.19 (0.86-1.65)

**Table III:** Inherited thrombophilia in the study population

		**Recurrent IVF failure group** **(N=96)**	**Healthy women** **(N=95)**	**p-value**	**OR (95% CI)**
Age (years)		36.16 ± 4.68	33.01± 5.06	0.59	
All thrombophilia’s		59 (61.5%)	31 (32.6%)	0.00	3.15 (1.74-5.70)
Factor V Leiden mutation		16 (16.7%)	5 (5.3%)	0.01	3.60 (1.26- 10.27)
MTHFR C677T mutation					
	-Homozygote	11 (11.5%)	1 (1.1%)	0.05	12.33 (1.55- 97.86)
	-Heterozygote	11 (11.5%)	11 (11.6%)		1.12 (0.45- 2.73)
Prothrombin 20210A mutation		9 (9.4%)	4 (4.2%)	0.16	2.35 (0.69- 7.92)
Hyperhomocysteinemia		7 (7.3%)	7 (7.4%)	0.98	0.98 (0.33- 2.93)
Protein C deficiency		4 (4.2%)	3 (3.2%)	0.71	1.33 (0.29- 6.12)
Protein S deficiency		4 (4.2%)	2 (2.1%)	0.42	2.02 (0.36- 11.30)
AT-III deficiency		2 (2.1%)	1 (1.1%)	0.57	2 (0.17- 22.43)
PAI-1 mutation					
	-Homozygote	2 (2.1%)	0	1.000	
	-Heterozygote	10 (10.4%)	10 (10.5%)		1.01 (0.40- 2.55)

MTHFR: methylene tetra hydro folate reductase.

AT-III: anti thrombin- III.

PAI-1: plasminogen activator inhibitor1.

## Discussion

Our results revealed a higher prevalence of inherited thrombophilia in women with repeated IVF failure compared with normal women. This fact reinforces the association of this pathology with vascular impairment and a consequent difficulty in embryo implantation. Factor V Leiden mutation and MTHFR C677T mutation were significantly more prevalent in IVF failure group, where other thrombophilia’s were not. Having at least one thrombophilia, mutation of factor V Leiden and homozygote form of MTHFR C677T mutation were risk factors for recurrent IVF failure. Recurrent pregnancy loss and pregnancy complications such as severe pre-eclampsia, fetal growth restriction and stillbirth are more common among patients with either inherited or acquired thrombophilia defect (13-15). A possible responsible mechanism could be the thrombosis of maternal vessels, which reduces the perfusion of intervillous space leading to placentation failure. Failures of implantation and early placentation of embryos in IVF may cause by similar mechanisms. Thrombosis in the placental vessels leads to hypo perfusion of the intervillous space and may cause placentation failure (13, 14). 

However, other mechanisms may be responsible too, like the damage of decidual or chorionic vessels, or reduction of trophoblast invasiveness (16, 17). Our finding is in agreement with those of who reported an association between IVF failure and an increase incidence of thrombophilia (7, 8). The mechanism by which thrombophilia affects recurrent pregnancy loss and IVF failure is yet undetermined. In line with our results Simur showed that a finding of at least one thrombophilia factor was more common in the group of women with implantation failure. However their result was not statistically significant, which could be the effect of their small study population (18). 

Factor V Leiden mutation and MTHFR C677T mutation were significantly more prevalent in IVF failure group, where other thrombophilia’s were not. There is however no consensus on type of inherited thrombophilia associated with repeated pregnancy loss. One study reported that mutations of the factor V Leiden and protrombin gene might have a role in implantation failure or in pregnancy loss after IVF (7). A review article suggest that factor V Leiden is more prevalent in women with IVF failure compared with fertile parous women or women who achieve a live birth after IVF. There is an overall 3-fold increased risk of IVF failure associated with factor V Leiden, which is significantly higher among the heterozygotes. 

In this review, the prevalence of MTHFR mutation in women with IVF failure was similar to controls overall and also when MTHFR homozygotes and heterozygotes were evaluated separately. However, MTHFR C677T mutation in our study was significantly more prevalent in IVF failure group, especially the homozygote form. In agreement with our study the presence of protein C, protein S, and anti-thrombin deficiencies were previously shown to be similar between women with and those without ART failure (19). Evaluation of thrombophilia’s may be an additional evaluation beside other host evaluations in patients with recurrent IVF failure. However, the relationship between ART failure and thrombophilia remains largely inconclusive. The result of our study is limited because of the case-control nature of the study and large prospective investigations are warranted to confirm this association before embarking in screening or intervention studies. 

## Footenote

***This article extracted from student thesis. (Elham Kheyrollahi)***
